# Does blood transfusion harm cardiac surgery patients?

**DOI:** 10.1186/1741-7015-7-38

**Published:** 2009-07-31

**Authors:** Gavin J Murphy

**Affiliations:** 1 Bristol Heart Institute, University of Bristol, Bristol Royal Infirmary, Bristol, UK

## Abstract

Over recent years there has been a substantial body of evidence demonstrating strong associations between transfusion and adverse outcomes, including myocardial, neurological and renal injury, in a range of clinical settings where transfusion is administered for reasons other than life-threatening bleeding. The strength of these associations across a range of clinical settings suggests that confounding and bias, the chief limitations of all observational studies, are unlikely to account for all of these observations. Given the wide range in transfusion rates in cardiac centres, with up to 100% of patients in some centres exposed to allogenic blood components, this evidence, albeit circumstantial, presents a strong argument for prospective randomised trials to attempt to determine, firstly, if transfusion causes adverse outcomes, and secondly, in which patient groups does the benefit of transfusion outweigh these risks? These issues are discussed in the context of an article published this month in *BMC Medicine*.

## Background

The development of modern blood services that enable large-scale allogenic red cell donation, storage and transfusion represents one of the greatest achievements of modern medicine. It has saved countless lives and is indispensable for the treatment of trauma patients and those with life-threatening haemorrhage. The early success of blood transfusion, occurring as it did during wartime, coupled with advances in storage techniques led to the widespread use of transfusion for indications where there is little, if any, evidence of efficacy. With the advent of evidence-based medicine over recent decades, coupled with the increasing prevalence of electronic prospective clinical databases, we have seen a large number of retrospective observational studies that appear to show an association between transfusion and adverse outcome in a range of clinical scenarios including cardiac surgery [1], general surgery [2], acute coronary syndromes [3] and in critical care [4], to name but a few. In fact, it would seem that, with very few exceptions [5] there is no evidence of efficacy beyond its use in haemorrhagic shock. The study by Rogers and colleagues, published this month in *BMC Medicine, is *a useful addition to the literature, and is strengthened by the large numbers of patients considered and the quality of the analysis performed [6]. In this case, transfusion was associated with a twofold increase in infection rates. Other studies have shown similar increases in cardiac, neurological and renal morbidity associated with transfusion in cardiac surgery [1,7].

The question as to whether transfusion causes adverse outcome in cardiac surgery remains unanswered, however. The Rogers study suffers from the limitations of all retrospective studies in that it cannot adjust for unmeasured confounders, in this case the use of aprotinin, or a measure of left ventricular function, or for the likelihood that there will have been bias in the prescribing of allogenic blood, with patients who are more ill being more likely to receive transfusions than those that are less ill. If we look for clinical evidence that transfusion causes adverse outcome, we can refer only to one study in adults; the Transfusion Requirements in Critical Care (TRICC) study [8]. This study, performed in a highly selected group of critical care patients compared a restrictive (7 g/dL) to a liberal transfusion threshold (10 g/dL). A total of 100% of those in the liberal group received an allogenic red blood cell (RBC) transfusion compared to 67% in the restrictive group. There was no difference in the primary endpoint (30-day mortality) between the groups, although there were some differences in secondary endpoints (myocardial infarction and pulmonary oedema), and in a secondary analysis in younger and less ill patients where there was a higher mortality in the liberal threshold group. This study has therefore not presented incontrovertible proof that transfusion is harmful. Moreover, the TRICC study is over 10 years old and blood storage techniques have changed since then, with the widespread introduction of leukodepletion in many countries. The applicability of these findings to other patient groups, such as cardiac surgery patients, is also unclear. It is unfortunate that our evidence base has not developed over the last the decade, particularly when one considers the strength of association between transfusion and adverse outcome in many studies. In the current study, exposure to allogenic blood components was associated with an almost fivefold increase in in-hospital mortality [6], and it is difficult to see how this could be attributed simply to confounding and bias. The potential economic burden of transfusion-associated morbidity is also considerable. In one study, after adjustment for confounding, transfusion of a single unit of RBC was associated with a 10% increase in hospitalisation costs [7]. Randomised trials that identify transfusion indicators are required if we are to target this resource to those patients that need it and, if nothing else, prevent waste of what is a valuable and scarce resource.

This brings us to the major question raised by the current study by Rogers and colleagues [7]: when is transfusion indicated, or rather when do the benefits outweigh the risks? Undoubtedly many patients undergoing cardiac surgery develop coagulopathic or surgical bleeding that is life-threatening and transfusion in this setting is clearly indicated. In cross-sectional studies in the UK the rate of re-thoracotomy for severe bleeding ranges from 3% to 10% [9]. Identifying other patients that benefit from transfusion is more difficult, although some severely anaemic patients without life-threatening bleeding will in all probability benefit from RBC transfusion. It is highly unlikely that 100% of patients undergoing cardiac surgery in any unit will need or benefit from transfusion however, as has been described in the current study. Even the lower confidence limit for transfusion rates in women in the current study, 72.5%, is high. The relative proportions of different types of component transfused were not stated, which limits our interpretation slightly; however, one could conclude that many of these transfusions were unnecessary. If transfusion does cause organ injury and adverse outcome, many of these patients suffered unnecessarily as a result. The answers to this however are that, firstly, where is the evidence of cause and effect, and secondly, what is or is not a necessary transfusion.

The strong associations between transfusion and adverse outcome and the variability in transfusion practice are strong arguments for a randomised trail in cardiac surgery. This should attempt to determine whether more transfusion is harmful than less transfusion. The TITRe2 (for 'Transfusion Indication Threshold Reduction on transfusion rates') trial, a multicentre randomised controlled trial of transfusion indication threshold reduction on transfusion rates, morbidity and healthcare resource use following cardiac surgery, is a UK National Institute for Health Research-funded study that will attempt to address this issue. A total of 2,000 patients in 10 UK cardiac centres will be randomised to either a restrictive transfusion threshold of 7.5 g/dL or a liberal threshold of 9 g/dL, with ischaemic and septic complications as a co-primary endpoint. The choice of thresholds is pragmatic, and reflects the range of haemoglobin over which the vast majority of transfusions are administered in clinical practice [10]. Whether these thresholds will reflect accurate transfusion indicators is another matter, and in all likelihood several trials will be required to define groups of patients where transfusion is beneficial. These trials are difficult to perform, not least because blood is transfused according to a medical theology where clinicians often have strong views as to when transfusions should be administered, despite the lack of available evidence, and resistance to enrolment is more common from clinicians, rather than patients. If, as suggested by Rogers and colleagues, transfusion harms more often than it helps however, we as clinicians have a duty to find out.

## Competing interests

GJM receives funding from the British Heart Foundation and the National Institute for Health Research to undertake research into blood conservation and safer blood transfusion practice.

## Pre-publication history

The pre-publication history for this paper can be accessed here:


